# Performance of the Cue COVID-19 point-of-care molecular test: insights from a multi-site clinic service model

**DOI:** 10.1128/spectrum.04064-22

**Published:** 2023-09-20

**Authors:** Anu Rebbapragada, Lane Cariazo, David Elchuk, Hossam Abdelrahman, Dang Pham, Jerusha Raveendraraj, Killol Chokshi, Nirochile Joseph, Elena Gouzenkova, Harpreet Gill, Peter Blecher

**Affiliations:** 1 FH Health, Toronto, Ontario, Canada; University of Mississippi Medical Center, Jackson, Mississippi, USA

**Keywords:** COVID, testing, point-of-care, coronavirus, molecular testing

## Abstract

The COVID-19 pandemic highlighted the critical need for rapid and accurate molecular diagnostic testing. The Cue COVID-19 Point-of-Care Test (Cue POCT) is a nucleic acid amplification test (NAAT), authorized by Health Canada and FDA as a POCT for SARS-CoV-2 detection. Cue POCT was deployed at a network of clinics in Ontario, Canada with *n* = 13,848 patrons tested between 17 July 2021 and 31 January 2022. The clinical performance and operational experience with Cue POCT were examined for this testing population composed mostly of asymptomatic individuals (93.7%). A head-to-head prospective clinical verification was performed between 17 July and 4 October for all POCT service clients (*n* = 3,037) with paired COVID-19 testing by Cue and RT-PCR. Prospective verification demonstrated a clinical sensitivity of 100% and clinical specificity of 99.4% for Cue COVID-19 POCT. The lack of false negatives and low false positive rate (0.64%), underscores the high accuracy (99.4%) of Cue POCT to provide rapid PCR quality results. Low error rates (cancellation rate of 0% and invalid rate of 0.63%) with the current software version were additionally noted. Taken together, these findings highlight the value of accurate molecular COVID-19 POCT in a distributed service delivery model to rapidly detect cases in the community with the potential to curb transmission in high-exposure settings (i.e., in-flight, congregate workplace, and social events). The insights gleaned from this operational implementation are readily transferable to future POCT diagnostic services.

**IMPORTANCE:**

This manuscript reports on the findings of a large asymptomatic population who underwent surveillance COVID testing on the Cue COVID-19 Point-of-Care Test (POCT). Review of test performance of this rapid molecular POCT, as compared to gold standard RT-PCR, is valuable to many audiences, including public health, emergency testing services, employers, and the general population of consumers who are seeking a user-friendly, accurate, cost-effective, and sustainable testing model for COVID screening. The findings from this operational experience also inform future models of POCT services beyond COVID.

## INTRODUCTION

Severe acute respiratory syndrome coronavirus (SARS-CoV-2) emerged as a pandemic threat in 2020 and continues to pose a global challenge with sustained waves of global infection, hospitalizations, and deaths due to respiratory infections (1). Due to the severity and heightened contagiousness of different SARS-CoV-2 variants, rapid and accurate testing is urgently required to reduce transmission and morbidity in high-exposure settings (i.e., healthcare, in-flight airline cabinet, congregate workplaces, and social gatherings) (2 – 5). Detection of SARS-CoV-2 RNA by nucleic acid amplification tests (NAATs), such as reverse transcription-polymerase chain reaction (RT-PCR), is the most common method (4). However, lab-based RT-PCR is disadvantaged by transportation to a central testing site, longer analytical process (often 4–6 h to perform specimen prep, extraction, and detection) and result analysis. These inherent limitations with lab-based testing hamper the objectives of rapid case detection, enactment of infection control precautions, and early treatment. Point-of-Care Testing (POCT) holds the potential for rapid detection of COVID-19 including screening of asymptomatic populations and the initiation of informed actions in a cost-effective and clinically efficacious manner (5 – 9).

**Fig 1:**
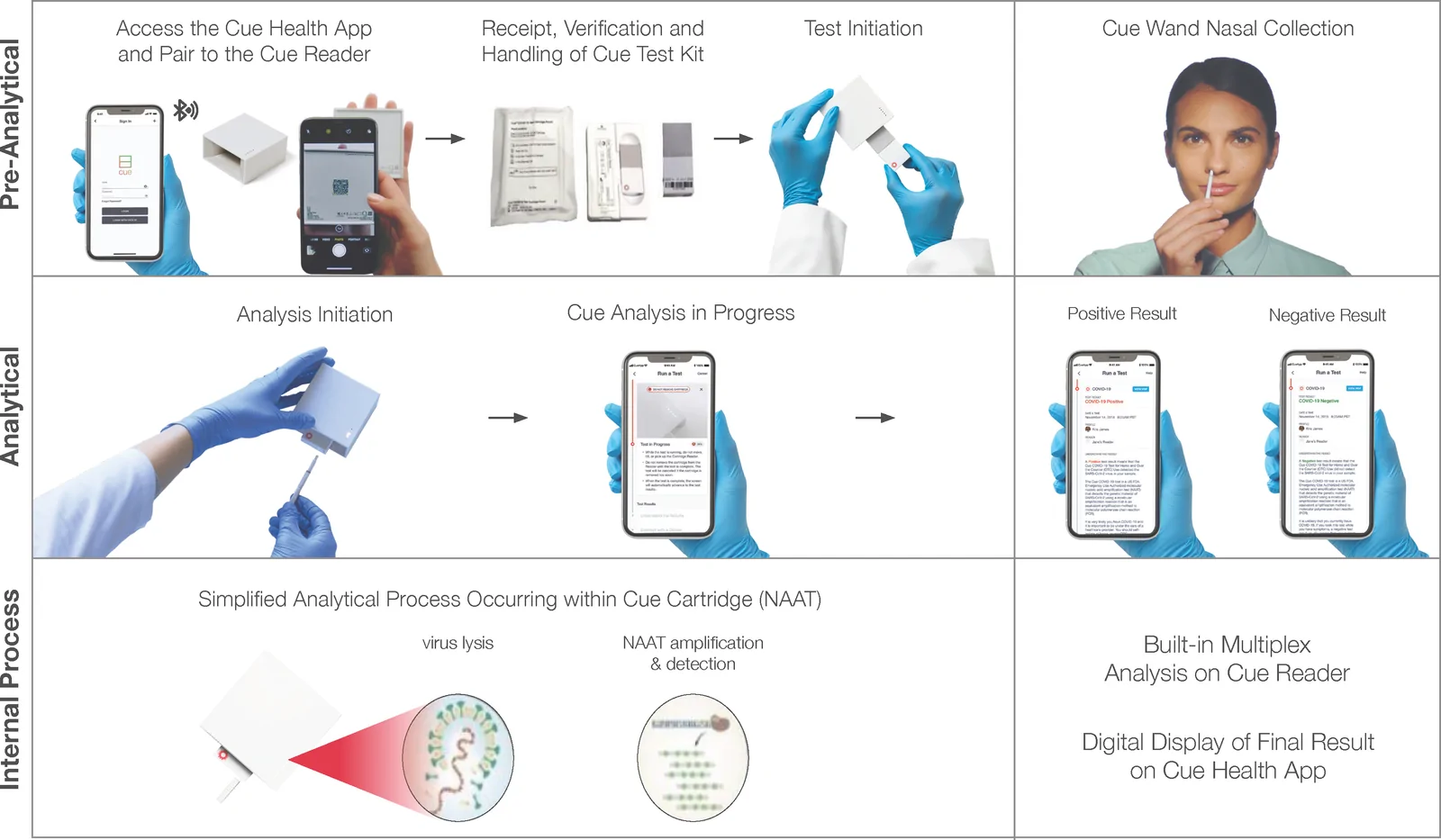
Cue COVID-19 Point-of-Care Test (POCT) workflow. The pre-analytical process begins by pairing the Cue Health Digital Application (App) with the Cue Reader, followed by insertion of the Cue reagent cartridge into the Cue Reader to pre-warm the test components and collection of a nasal specimen with the Cue Wand. Once the Cue Health App indicates the test components are ready, the Cue wand specimen is inserted into the warmed Cue cartridge to initiate the analytical process which takes ~20 min. The molecular testing process (lysis, isothermal nucleic acid amplification, and multiplex detection) is integrated within the Cue cartridge and reader. Target signals are immediately analyzed and final interpreted results (positive, negative, and invalid) are transmitted wirelessly to the paired Cue Health App for secure digital reporting. Results may be viewed directly by the test taker on the App and on a dashboard by service providers to track aggregate result metrics. (Reproduced from Cue marketing materials with permission)

The Cue COVID-19 molecular test (Cue POCT) is one of a handful of rapid molecular POCT that have received emergency use authorization (EUA) by US Food and Drug Administration (FDA) and Health Canada Emergency Interim Order (IO) to detect SARS-CoV-2 (6, 10, 11). Cue COVID-19 is an isothermal nucleic acid amplification test for qualitative multiplex detection of the Nucleocapsid (N) gene of SARS-CoV-2 and, human RNaseP as an endogenous control to verify specimen adequacy and proper assay function. Lysis, amplification, and detection take place within the Cue cartridge with final results (positive, negative, or invalid) available in about 20 min. Amplification of targets permits the binding of conjugates on the forward primers (biotin on N-gene or digoxigenin on RNaseP) and reverse primers (horseradish peroxidase) to produce electrochemical signals for detection. The Cue COVID-19 test kit includes a Cue wand to collect a nasal swab sample, a single-use test cartridge, and a multi-use Cue reader device that wirelessly connects to the Cue Health App to display results (Fig. 1). This report describes the performance of the Cue POCT at a network of clinics in Ontario, Canada providing COVID-19 screening services to asymptomatic individuals for the purposes of reassurance, travel permission, or entry into workplace and congregate events. The insights gleaned from test sensitivity, specificity, and operational parameters can inform the implementation of future POCT diagnostic services.

## MATERIALS AND METHODS

### Testing centers

Specimen collection and Cue COVID-19 POCT were performed at a network of FH Health clinics in Ontario, Canada by registered healthcare providers (nurses or practical nurses) and RT-PCR testing was conducted at the FH Health Laboratory, licensed by the Ontario Ministry of Health and accredited under ISO 15189 standards. Clinic sites included densely populated urban locations, as well as suburban settings. Patrons booked a clinic appointment digitally (via web or app) and upon arrival at the clinic, FH Health administrators verified patron identity, and collected information on symptoms and vaccination status. The data presented in this manuscript represent a prospective verification of test performance of the Cue COVID-19 Point-of-Care Testing system versus RT-PCR leveraging results gathered from provision of routine testing services. This study methodology was reviewed by Advarra Review Board and was considered to be exempted. They noted the framework of this comparison (where it was conducted, information provided to paying patrons, anonymized data analysis, and report) fulfilled the criteria for Exemption under the Common Rule (45 CFR subpart A).

### Cue COVID-19 molecular test at FH Health clinics

The Cue COVID-19 POCT system was utilized across seven FH Health clinics for patrons who booked the “Express” service with results in Turnaround Time (TAT) of 1–6 h. Cue COVID-19 POCT was performed as per the approved Instructions for Use under Health Canada interim order [IFU, (11)] by collecting a deep nasal specimen with the Cue wand, insertion into a Cue cartridge readied for testing by pre-heating on a Cue device and reviewing results on the paired Cue Health app (Fig. 1). The entire Cue POCT workflow requires approximately 25 min (3 min pre-analytical and 22 min analytical time).

### Laboratory RT-PCR tests

After collection of the anterior nasal sample for Cue testing with the Cue wand, nurses collected a bilateral deep nasal specimen with an FDA EUA collection device (Bioer, Hangzhou Bioer Technology Co, Ltd) using a sterile nylon fiber non-flocked swab and placed it into a tube containing 2 mL molecular transport media with guanidine for transportation to the lab for RT-PCR testing. RT-PCR specimens were tested within 6–12 h of collection on one of the following RT-PCR assays: (i) Osang GeneFinder COVID-19 (FDA EUA and Health Canada IO), (ii) ThermoFisher TaqPath COVID-19 Combo Kit (FDA EUA and Health Canada IO) or, (iii) New England Biolabs Luna One Step SARS-CoV-2 RT-qPCR Multiplex Assay kit. TAT for FH Health’s lab-based RT-PCR testing offered results within 12 or 24 h, as “same day” or “next day” services.

Three different assays were validated to provide flexibility and insulate against supply chain constraints. Assays were utilized based on current reagent stock levels and workflow. RT-PCR specimens were primarily tested with the FDA EUA Osang GeneFinder COVID-19 assay (Osang). When Osang reagents were not available, RT-PCR specimens were tested either with the FDA EUA ThermoFisher TaqPath COVID-19 Combo Kit (TaqPath) or the New England Biolabs Luna SARS-CoV-2 RT-qPCR Multiplex Assay kit (Luna).

Samples for all assays were tested on a 96-well PCR plate with three external quality control wells on each plate. Positive SARS-CoV-2 detection was reported when two or more viral targets were detected with amplification Ct values under 37 for the TaqPath assay or Ct values under 40 for the Osang and Luna assays. Indeterminate SARS-CoV-2 detection was reported when only one viral target was detected with Ct values under 37 for the TaqPath assay or Ct values under 40 for the Osang and Luna assays. The amplification curves for all positive and indeterminate samples were evaluated by certified medical lab technologists. Samples reported as negative fulfilled the criteria for internal process control detection (MS2 on TaqPath) or human RNaseP (GeneFinder and Luna assays) indicating proper specimen collection, operator setup, and assay system performance when viral target genes were not detected. Samples with no RNaseP amplification were reported as Invalid to indicate an issue with specimen collection and to trigger recall of the patron for repeat collection and testing. RT-PCR assays were verified to detect two copies of SARS-CoV-2 per reaction. The Osang GeneFinder and New England Biolabs Luna assays demonstrated enhanced analytical sensitivity to reliably detect one copy of SARS-CoV-2 per reaction.

### Result reporting

All results were digitally provided directly to patrons as a PDF report via FH Health’s proprietary Clinic and Laboratory Information System. The accuracy, reliability, PHIPPA compliance, and security of digital reporting were verified prior to deployment and met the requirements of ISO 15189 accreditation standards.

### Cue POCT performance verification prior to implementation

#### Analytical sensitivity

As per the IFU analytical sensitivity or limit of detection (LoD) of the Cue POCT was established by testing known amounts of purified viral culture (diluted in negative clinical nasal specimens) and claims a LoD of 20 copies when the Cue sample wand is used for specimen collection and direct testing on the Cue device (10, 11). This claimed LoD was verified with a contrived panel of positive specimens prepared with Microbix REDx FLOQ swab-based positive controls for the wild type and five variant strains of SARS-CoV-2 including, B.1.1.7-Alpha, B.1.351-Beta, B.1.617-Delta, P.1-Gamma, and B.1.1.529-Omicron. Microbix REDx FLOQ controls contain the whole genome of each SARS-CoV-2 strain (estimated 35,000 copies/swab) and include human fibroblasts to permit evaluation of the specimen adequacy target detection (human RNaseP) used in the Cue POCT. A dilution series of contrived specimens (for wild type and five variants of SARS-CoV-2) was prepared in 1 mL viral transport medium (Microbix DxTM) to attain a final copy number of 100, 20, 10, 5, 1, 0.5, and 0.2 SARS-CoV-2 per Cue wand to assay the reportable range and verify the LoD. A Cue sample wand was dipped into each dilution to saturate sample absorption (approximately 50 mL absorbed per wand) and tested on the Cue device. Results verified the claimed LoD of 20 copies per Cue wand for viral target gene detection.

#### Accuracy and precision

Since residual RT-PCR specimens collected in molecular transport media could not be used to perform the Cue POCT (due to the presence of guanidine, which interferes with the Cue test reagents), accuracy was verified by testing a contrived panel of 20 positive specimens (prepared with the Microbix REDx FLOQ Positive Control Swab) and 20 negative specimens (collected with the Cue wand from 20 COVID-19 negative staff members). Cue POCT precision was verified by testing at least three positive and three negative samples in duplicate for inter-device and inter-operator reproducibility. The Cue POCT system (reagents and reader device) met the acceptance criteria for accuracy and precision with 100% concordant results.

## RESULTS

### Clinical performance of Cue COVID-19 POCT

#### Demographics of the Cue COVID-19 testing population

Cue COVID-19 results (*n* = 13,848) generated from 17 July 2021 to 31 January 2022 encompass the entire testing period described in this report and reflect the broad age range receiving POCT services at FH Health clinics across Ontario (Table 1). FH Health clinic attendees were primarily asymptomatic (*n* = 12,986, 93.8%), presenting for paid COVID-19 testing services (i.e., travel, employment, and/or health “reassurance”) (Table 2).

**TABLE 1:** Age demographics of the entire Cue COVID-19 test population from 17 July 2021 to 31 January 2022 (*n* = 13,848 individuals)

Birth year	Category	Number of patients
2018–2021	Baby (0–3)	191
2004–2017	Youth (4–17)	1,027
1982–2003	Adults (18–39)	5,637
1962–1981	Middle aged (40–59)	5,060
<1961	Seniors (60+)	1,933
	Total	13,848

**TABLE 2:** Cue COVID-19 results by symptom status for the entire testing period from 17 July 2021 to 31 January 2022 (*n* = 13,848 individuals)

Symptom status	Total individuals, number, proportion	Cue positive, number, proportion	Cue negative, number, proportion
Symptomatic	*n* = 863, 6.23%	*n* = 142, 16.4%	721, 5.55%
Asymptomatic	*n* = 12,986, 93.8%	*n* = 726, 83.6%	12,260, 94.5%
Total tests	13,849	868	12,981

#### Specimen disposition for paired Cue COVID-19 and RT-PCR testing

A total of 3,368 tests were performed on the Cue COVID-19 POCT from 17 July to 4 October 2021. From this set, results from *n* = 3,037 patrons with ages ranging from under 1 year to over 60 years were included for comparative analysis with evaluable parallel Cue and lab-based RT-PCR results (Table 3).

**TABLE 3:** Age demographics of the test population from 17 July to 4 October 2021 with paired COVID-19 Cue and RT-PCR tests (*n* = 3,037 individuals)

Birth year	Category	Number of patients
2018–2021	Baby (0–3)	57
2004–2017	Youth (4–17)	259
1982–2003	Adults (18–39)	1,197
1962–1981	Middle aged (40–59)	1,090
<1961	Seniors (60+)	434
	Total	3,037

Among the 3,368 tests performed, *n* = 331 tests were excluded from comparative analysis due to invalid or canceled Cue POCT results and lack of a parallel RT-PCR specimen. One hundred fifty-one tests had an initial invalid (*n* = 116) or canceled (*n* = 35) result representing a combined error rate of 4.48%. Re-testing these invalid and canceled specimens as per the IFU yielded 135 valid results. Of the 116 invalid Cue tests, 6 could not be resolved and were sent to the laboratory for PCR testing. Twenty-five of the canceled Cue tests produced a negative test upon re-testing. The remaining 10 canceled tests could not be resolved, so RT-PCR samples were sent to the laboratory to obtain a valid result. Parallel samples for RT-PCR testing could not be collected from 180 individuals, including two Cue-positive patrons, either because the patron could not return to the clinic or refused a parallel sample collection.

Among the evaluable paired results (2,955 SARS-CoV-2-negative and 82 SARS-CoV-2-positive), a majority were derived from self-reported asymptomatic patrons (*n* = 2,998/3,037, 98.7%). Highly concordant positive results were obtained between Cue and RT-PCR (*n* = 61/63, 96.8%) among the asymptomatic. Symptomatic clinic POCT patrons represented a smaller proportion of paired results (*n* = 39/3,037, 1.28%) and positive detections (*n* = 2/63, 3.17%). Refer to Table 4.

**TABLE 4:** COVID-19 test results by symptom status for paired Cue and RT-PCR tests from 17 July to 4 October 2021 (*n* = 3,037 individuals)

Symptom status, Number (proportion)	True negative both cue and RT-PCR negative	True positive both cue and RT-PCR positive	False positive Cue positive but RT-PCR negative	False negative Cue negative but RT-PCR positive
Asymptomatic *n* = 2,998 (98.7%)	2,918 (98.7%)	61 (96.8%)	19 (100%)	0
Symptomatic *n* = 39 (1.28%)	37 (1.25%)	2 (3.17%)	0 (0%)	0
Total *n* = 3,037 (100%)	2,955	63	19	0

As of 5 October 2021, Cue test verification passed technical review by accreditation. After this point, parallel testing with RT-PCR was only performed for Cue patrons who tested positive or experienced two consecutive invalid or canceled results. During the period from 5 October 2021 to 31 January 2022, an additional *n* = 10,811 Cue tests were performed, bringing the total number of Cue COVID-19 results for the entire period (17 July 2021 to 31 January 2022) to *n* = 13,848.

#### Cue COVID-19 test performance characteristics

The Cue COVID-19 demonstrated superb clinical sensitivity (100%, *n* = 63/63) and high clinical specificity (99.4%, *n* = 2,955/2,974) (Table 5). Cue demonstrated 99.4% concordance with RT-PCR testing, providing 100% negative predictive value (confidence in negative results) and high positive predictive value in the context of rising disease prevalence (97% when 20% prevalence).

**TABLE 5:** Clinical test performance characteristics of Cue COVID-19 versus RT-PCR during the parallel testing period from 17 July to 4 October 2021 (*n* = 3,037 individuals)

Cue result	RT-PCR positive	RT-PCR negative	Total
Cue positive	63	19	82
Cue negative	0	2,955	2,955
Total number of test results			3,037

Test Performance Metric for COVID-19 Detection	Cue POCT versus RT-PCR (number, proportion)
Sensitivity	*n* = 63/63, **100%**
Specificity	*n* = 2,955/2,974, **99.4%**
False positive rate	*n* = 19/2,974, **0.64%**
Accuracy	*n* = 3,018/3,037, **99.4%**
False negative rate	*n* = 0/63, **0%**
Positive predictive value	**76.8%** (if 2% prevalence) **97%** (if 20% prevalence)
Negative predictive value	**100%**

#### Comparison of reportable ranges between Cue COVID-19 and RT-PCR

**Fig 2:**
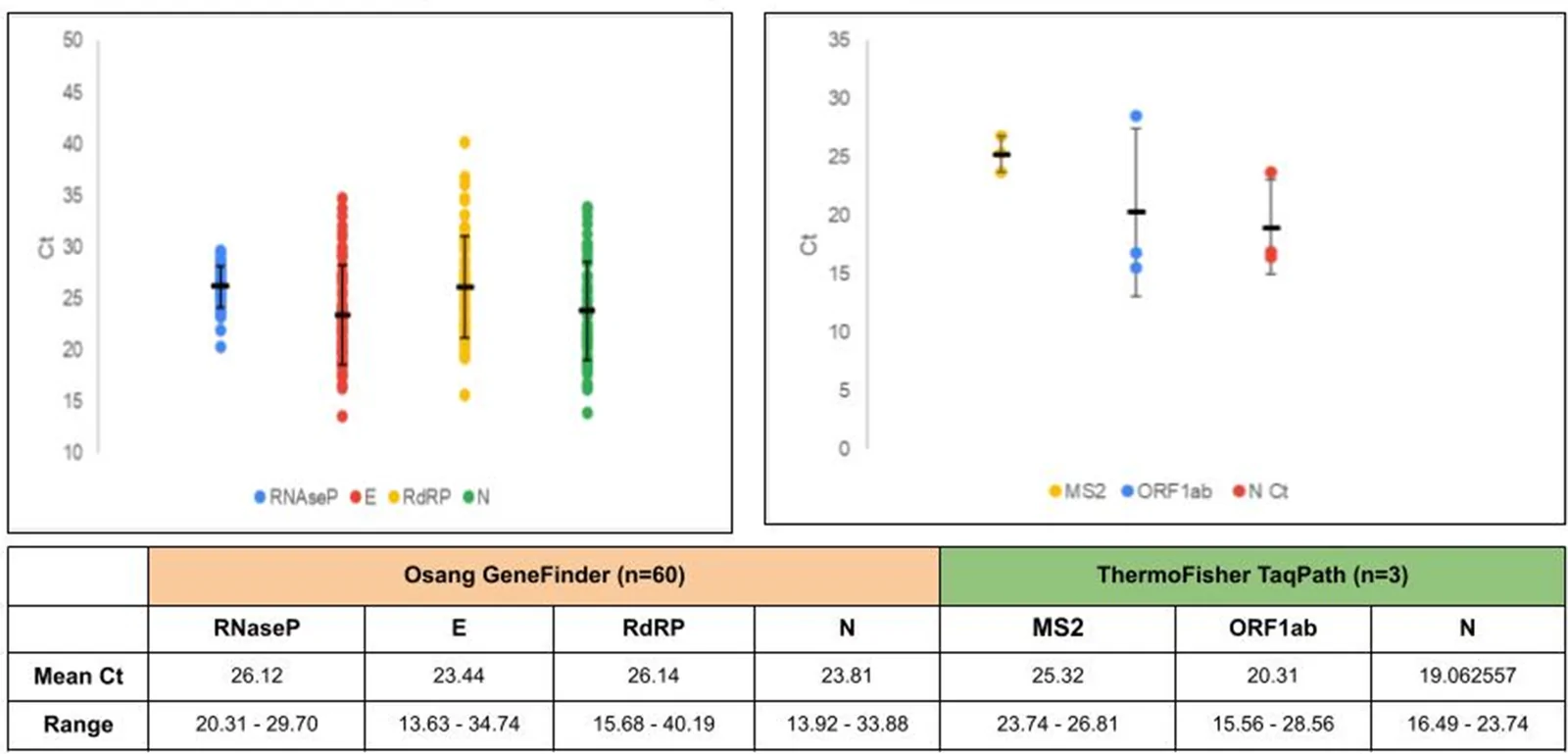
Cycle threshold (Ct) distribution for Cue true positives for the paired testing period from 17 July to 4 October 2021 (*n* = 63 results). (**A**) Osang (*n* = 60 results). (**B**) ThermoFisher TaqPath (*n* = 3 results).

**Fig 3:**
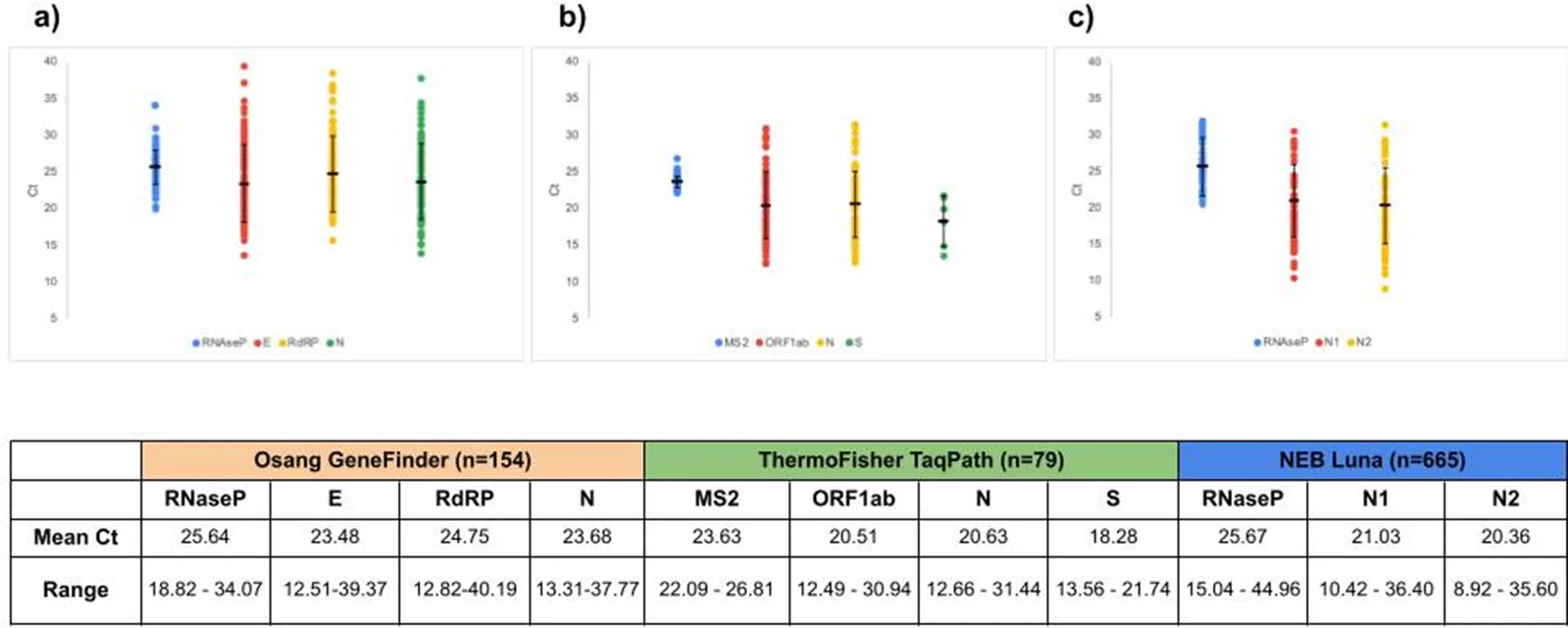
Cycle threshold (Ct) distribution for Cue true positives for the entire testing period from 17 July 2021 to 31 January 2022 (*n* = 898 results). (**A**) Osang (*n* = 154 results). (**B**) ThermoFisher TaqPath (*n* = 79 results). (**C**) Luna (*n* = 665 results).

The range of RT-PCR cycle threshold (Ct) values obtained on specimens with Cue positive detections indicate a broad reportable range for paired samples that underwent Cue and PCR testing from July to October 2021 (Fig. 2) and the entire period till 31 January 2022 (Fig. 3). RT-PCR Ct values for viral genes ranged from 13 to high 30s, demonstrating that Cue POCT is able to detect virus throughout the natural course of infection, i.e., the natural history of infection from early symptom onset (post-exposure, early infection phase), peak viremia (high viral replication phase), and declining titers of viral load (recovery phase). Of note, five samples that tested positive by Cue had high mean Ct values (range: 34.5–38.3) with only a single gene detected by RT-PCR. These samples were categorized as “indeterminate” as per the laboratory reporting algorithm and recalled for follow-up testing. One patron did not return for testing. Two patrons tested positive when RT-PCR was performed on a new specimen collected a day after the original Cue positive result and two patrons had a recent history of prior infection indicating residual viral shedding during convalescence. Based on the observations that Cue is able to detect SARS-CoV-2 in samples with higher RT-PCR Ct values for the viral target, it is possible that Cue can identify infection in individuals with lower viral load, either at the early stages of a new infection or during the convalescent stage after symptoms have cleared. However, the higher RT-PCR Ct values may be caused by external processing factors beyond the specimen’s viral copy number; factors, such as specimen collection, pre-analytical handling, extraction, and/or the presence of interfering substances, may have raised RT-PCR Ct values.

#### Discordant results between Cue COVID-19 and RT-PCR

**Fig 4:**
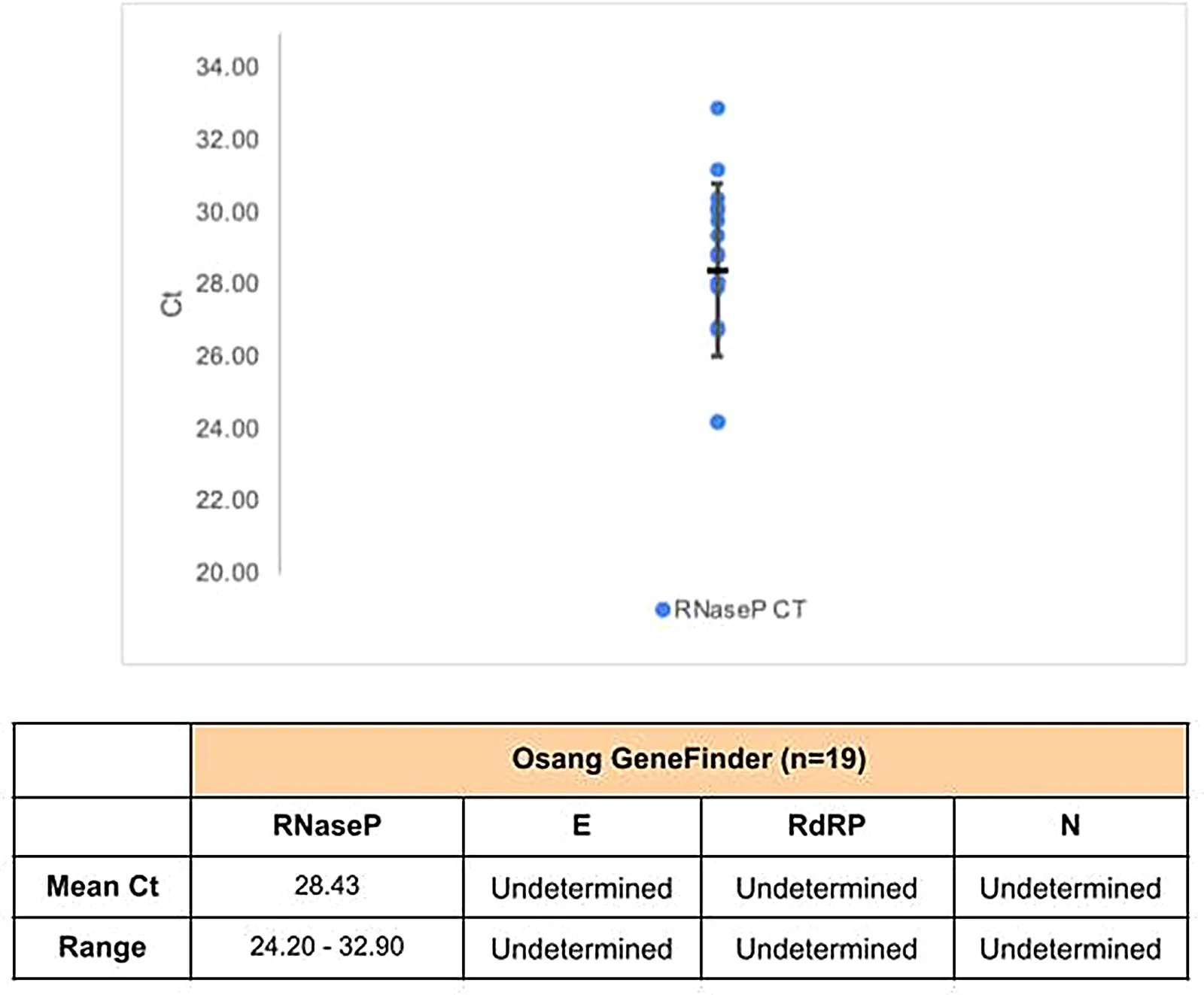
Cycle threshold (Ct) distribution for Human RNaseP endogenous control detection in Cue False Positives for the paired testing period from 17 July to 4 October 2021 (*n* = 19 results).

The parallel testing data revealed a false positive rate of 0.64% (*n* = 19/2,974 results) for the period of 17 July to 4 October 2021. All samples with discordant Cue POCT and RT-PCR results (i.e., Cue positive but RT-PCR negative) were re-tested by RT-PCR to confirm the validity of the negative RT-PCR result. All Cue POCT false positive results had valid human RNaseP detection by RT-PCR (Fig. 4) and the three Quality Control wells included on each PCR plate (extraction control, positive control, and negative control) had valid/pass results indicating the proper performance of RT-PCR reagents, equipment, and operators. As of 5 October 2021, parallel RT-PCR testing was only performed on patrons who tested positive with Cue COVID-19. Discordant results (*n* = 29/13,848, 0.21% with Cue positive and RT-PCR negative) were detected among the *n* = 13,848 results generated during the entire period from 17 July 2021 to 31 January 2022 with the majority identified in asymptomatic individuals (*n* = 27/29) (Table 6)**.** Cue R&D attributes the small proportion of false positives to aberrant primer-primer interactions, an infrequent but possible artifact in nucleic acid amplification technologies and/or internal mechanical errors that could have affected buffer flow. Their extensive *in silico* BLAST analysis of Cue COVID-19 primers and probe revealed zero cross-reactive hits (i.e., no sequence homology with other respiratory pathogens) for both the forward and reverse primers. The probe displayed alignment only with SARS-1 and SARS-2 with greater than 50% homology. Cue’s internal “wet lab” cross-reactivity testing also demonstrated zero false positive detection of multiple respiratory pathogen strains tested including 20 replicates of Rhinovirus, Adenovirus, and *Staphylococcus epidermidis.* The data from the latest R&D cross-reactivity testing will be included in the updated IFU submitted for FDA *de novo* authorization.

**TABLE 6:** Paired COVID-19 results with Cue and RT-PCR for the entire testing period from 17 July 2021 to 31 January 2022 (*n* = 13,848 individuals) by symptom status

Cue result	Concordant RT-PCR positive	Discordant RT-PCR negative	Total tests
Cue positive	*n* = 839 Symptomatic: *n* = 140 Asymptomatic: *n* = 699	*n* = 29 Symptomatic; *n* = 2 Asymptomatic: *n* = 27	868
Cue negative	n/a^*a*^		12,980
Total test results			13,848

^*a*^ As of 5 October, only individuals with positive, canceled or invalid Cue results were tested by RT-PCR.

#### Cue COVID-19 error rates

A total of *n* = 14,331 tests were performed on the Cue COVID-19 from 17 July 2021 to 31 January 2022 with 95.86% valid results (*n* = 13,739 tests with *n* = 12,585 negative, 87.81% and *n* = 1,154 positive, 7.95%) (Table 7).

**TABLE 7:** Cue COVID-19 results or test outcome from 17 July 2021 to 31 January 2022 (*n* = 14,331 tests)

Result or test outcome	Number	Rate
Valid negative	12,585	87.81%
Valid positive	1,154	7.95%
Invalid due to sample or operator error	435	3.11%
Canceled; device error	157	1.13%
Cue Health reporting app error	0	0%
Total	14,331	

The Cue Health App implemented serial firmware updates starting in mid-December 2021 which reduced the occurrence of false positives by improving the detection of mechanical issues related to wash buffer flow failure. The safety update detects failure of this important process step and cancels the test instead of reporting a false positive result. The false positive rate was calculated for testing performed with three different firmware versions on the Cue Health App. Upon implementation of serial firmware updates, the false positive rate among all samples tested progressively decreased from 0.28% (v.1.0.0) to 0.096% (v.1.5.0/v.1.5.1) to 0% (v.1.5.2). There was a threefold decline in false positives upon implementation of the second firmware version, representing a statistically significant reduction (*P* = 0.026) (12). In light of these observations with the firmware updates, cross-reactive detection of another respiratory pathogen is less likely to be the underlying reason for false positives.

Errors due to test cancellation or invalid results were also reduced with successive rounds of software upgrades (Table 8). Invalid errors are mainly due to improper specimen collection (inadequate sample obtained), operator errors in process or cartridge chemistry issues which can potentially compromise human RNaseP amplification. Invalid rates decreased from 3.85% to 2.17% to 0.68% corresponding to firmware upgrades from versions 1.0.0 to 1.5.0 to 1.5.2, respectively (Table 8) and re-training of healthcare workers on deep nasal specimen collection with the Cue wand, test initiation, and operation of the Cue device.

**TABLE 8:** Cue COVID-19 false positive, invalid, and canceled rates with different Cue Health App firmware updates performed from July 17 2021 to January 31 2022 (*n* = 14,331 tests)

Test outcome	Cue App firmware version updates		
	Version 1.0.0 (17 July to 14 December 2021), *n* = 8,400 tests	Version 1.5.0/1.5.1 (15 December to 19 January 2022), *n* = 5,199 tests	Version 1.5.2 (20 January to 31 January 2022), *n* = 732 tests
False positive	*n* = 24, 0.28%	*n* = 5, 0.096%	*n* = 0, 0%
Invalid	*n* = 323, 3.85%	*n* = 113, 2.17%	*n* = 5, 0.68%
Canceled	*n* = 123, 1.46%	*n* = 35, 0.67%	*n* = 0, 0%

Cancellation errors are due to safety check error (test cancels early upon initiation of process), component error, or lack of detection of flow over sensor after amplification. The cancellation error rate fell from 1.46% to 0.67% to 0% with serial software upgrades from versions 1.0.0 to 1.5.0 to 1.5.2, respectively (Table 8).

#### Turnaround time operational metrics

Fewer than 1% of the study participants had test results delayed by over 2 h (120 min) due to test cancellation or repeated invalid results, thus minimizing the necessity to collect a new specimen or direct samples to the lab for RT-PCR testing (Table 9).

**TABLE 9:** Cue COVID-19 Turnaround Time (TAT) for results reported from 17 July 2021 to 31 January 2022 (*n* = 13,848 reported results)

TAT	Number	Rate
<30 min	13,406	96.80%
<60 min	404	2.92%
>120 min	38	0.27%
Total	13,848	

## DISCUSSION

This study sought to ascertain the real-world efficacy of a COVID-19 screening program using the Cue POCT compared with RT-PCR in predominantly asymptomatic individuals. No false negatives were noted in this study cohort suggesting that the highly sensitive Cue POCT can rapidly and accurately detect cases. The high sensitivity of Cue detection has a significant utility within screening programs for any large group gatherings (i.e., workplace, congregate events, residences such as long-term care homes, dormitories, etc.) by rapidly identifying asymptomatic cases and promoting informed isolation and cohorting procedures which reduce the likelihood of transmission, and promote safe gatherings and business continuity. Additionally, the specificity of Cue POCT indicates that false positives are infrequent (high positive predictive value), decreasing the risk of unnecessary quarantine and the need for lab-based RT-PCR confirmatory testing which can inherently delay social gatherings (13).

Early and accurate detection, particularly for the Omicron and more recent variants, has been inconsistent by antigen testing as reviewed in multiple commentaries (14 – 16). These observations are corroborated by findings described in Adamson et al. (17), as discordant antigen test results were frequently noted in both symptomatic and asymptomatic individuals when compared to molecular testing. The sensitivity of the Cue COVID-19 POCT indicates it is an accurate proxy for traditional lab-based PCR testing with the added advantage of rapid results. The ability to accurately detect low viral load during the early stages of the natural history of infection could also provide a cost-effective solution that avoids the necessity for serial testing on consecutive days (14, 18, 19). Taken together, these performance features position the Cue COVID-19 POCT as an important tool in the arsenal for asymptomatic screening with high accuracy, improved resource utilization, and faster results. The performance characteristics also confirm that the real-world clinical sensitivity of the Cue COVID-19 POCT for SARS-CoV-2 wild type and variants was much closer to gold standard RT-PCR testing.

The findings of this study can further be extrapolated to considerations of “Test to Treat” initiatives (20). As the pandemic has evolved, novel treatment options have become available, including Molnupiravir and Paxlovid, as well as monoclonals. A new feature of Cue allows the user to directly connect to a platform for treatment, interface with a healthcare provider, receive a prescription, and have it delivered directly to their residence (21). The inclusion of Cue POCT in “Test to Treat” initiatives holds the potential for synergistic benefits by improving time to diagnosis, accelerating clinical management, minimizing delays in treatment initiation and ultimately promoting treatment effectiveness with rapid viral clearance, reduced complications, and lower hospitalization rates. Narrowing the time interval from test to treatment accelerates how quickly the patient can begin convalescence and return to work, which reduces overall economic stress, and has particular benefit to at-risk communities where other health and community resources, including testing, can be limited.

While the Cue COVID-19 test has a claim for home use and “over the counter” sale in the United States under the FDA emergency use authorization, this study was not able to evaluate the performance with specimens self-collected by individuals without formal training; Health Canada authorization only permitted collection by trained healthcare workers. In contrast to healthcare professionals, untrained individuals may have a learning curve to perform reliable self-swabbing which could in turn impact downstream test performance. However, Cue provides an in-app video, step-by-step visual mobile app instructions, and printed instruction sheets to ensure appropriate sampling methods, and there is ample data with other systems to show self-swabbing is reliable, safe, and comparable to healthcare worker collection. In addition, the detection of variants was not confirmed in clinical specimens by viral culture and sequencing. However, testing of contrived specimens confirmed that the Cue COVID-19 POCT was able to detect wild type and five variant strains including, B.1.1.7-Alpha, B.1.351-Beta, B.1.617-Delta, P.1-Gamma, and B.1.1.529-Omicron.

Other limitations of the current study include the lack of direct comparison to antigen testing, detailed cost-benefit, and health outcomes analysis. However, when factoring the full operational costs of RT-PCR testing (specimen collection, transportation, laboratory personnel, facilities, reagents, and equipment) and serial antigen testing on consecutive days, the cost of Cue COVID-19 testing provides an attractive value proposition. Additionally, Cue POCT did not incur many of the logistical costs or operational burdens experienced by lab RT-PCR; delays in lab results compromise early initiation of treatment and associated health outcome benefits which incur complex health system costs that are harder to enumerate.

This study represents one of the largest sets of data comparing lab-based RT-PCR to point-of-care molecular COVID-19 testing in a real-world population of largely asymptomatic individuals. The results of the current study underscore the success of Cue POCT in a community setting and the potential for broader population benefits, particularly in congregate settings, and in high-risk populations. Early case identification, prior to symptom onset, has the potential to curtail transmission and limit the immense burden of disease complications, healthcare costs and socio-economic disruptions. The accuracy of Cue results, the ease of use of the digitally enabled POCT device and the digital result readout on the mobile Cue Health app provide a cost-effective testing alternative to current methodologies. Additionally, the high sensitivity and specificity of Cue COVID-19 POCT provide actionable results with confidence to employers and businesses seeking to proactively identify cases and prevent further spread of COVID. The “Test to Treat” feature of Cue POCT represents novel capabilities that integrate diagnostics with electronic prescription and virtual physician consultation through a mobile application, dramatically improving clinical management (reduced time to treatment) for COVID-19, and serves as a model for other communicable diseases.

## PRESENTED AT

Canadian Association for Clinical Microbiology and Infectious Diseases (CACMID) conference in Vancouver, BC, Canada in April 2022

## References

[1] Centres for Disease Control . 2022. COVID-19 data tracker. Available from: https://covid.cdc.gov/covid-data-tracker/#datatracker-home

[2] Beach LA , Fung AWS , Knauer MJ , Shaw JLV , Taher J . 2021. Rapid COVID-19 testing: speed, quality and cost. Can you have all three? Clin Biochem 95:13–14. 10.1016/j.clinbiochem.2021.05.009 PMC8149163 34048775

[3] Vandenberg O , Martiny D , Rochas O , van Belkum A , Kozlakidis Z . 2021. Considerations for diagnostic COVID-19 tests. Nat Rev Microbiol 19:171–183. doi:10.1038/s41579-020-00461-z 33057203 PMC7556561

[4] Udugama B , Kadhiresan P , Kozlowski HN , Malekjahani A , Osborne M , Li VYC , Chen H , Mubareka S , Gubbay JB , Chan WCW . 2020. Diagnosing COVID-19: the disease and tools for detection. ACS Nano 14:3822–3835. doi:10.1021/acsnano.0c02624 32223179

[5] May L , Tran N , Ledeboer NA . 2021. Point-of-care COVID-19 testing in the emergency department: current status and future prospects. Expert Rev Mol Diagn 21:1333–1340. doi:10.1080/14737159.2021.2005582 34758686 PMC8631689

[6] Donato LJ , Trivedi VA , Stransky AM , Misra A , Pritt BS , Binnicker MJ , Karon BS . 2021. Evaluation of the cue health point-of-care COVID-19 (SARS-CoV-2 nucleic acid amplification) test at a community drive through collection center. Diagn Microbiol Infect Dis 100:115307. doi:10.1016/j.diagmicrobio.2020.115307 33571863 PMC7785428

[7] Hansen G , Marino J , Wang Z-X , Beavis KG , Rodrigo J , Labog K , Westblade LF , Jin R , Love N , Ding K , Garg S , Huang A , Sickler J , Tran NK . 2021. Clinical performance of the point-of-care cobas liat for detection of SARS-CoV-2 in 20 minutes: a multicenter study. J Clin Microbiol 59:e02811-20. doi:10.1128/JCM.02811-20 33239382 PMC8111162

[8] Valera E , Jankelow A , Lim J , Kindratenko V , Ganguli A , White K , Kumar J , Bashir R . 2021. COVID-19 point-of-care diagnostics: present and future. ACS Nano 15:7899–7906. doi:10.1021/acsnano.1c02981 33984237

[9] Loeffelholz MJ , Tang YW . 2021. Detection of SARS-CoV-2 at the point of care. Bioanalysis 13:1213–1223. doi:10.4155/bio-2021-0078 34289741 PMC8297542

[10] Cue COVID-19 test instructions for use. 2022. FDA EUA versions. Available from: https://www.fda.gov/media/138826/download https://www.fda.gov/media/146470/download

[11] Cue COVID-19 test instructions for use. 2022. Health Canada Interim Order Canadian version. Available from: https://www.cuehealth.com/documentation/home-otc/canada/Cue_COVID-19_OTC_Test_Instructions_For_Use_(IFU)_Canada.pdf

[12] MedCalc Software Ltd . 2023. Comparison of proportions Calculator. Available from: https://www.medcalc.org/calc/comparison_of_proportions.php

[13] Skittrall JP , Wilson M , Smielewska AA , Parmar S , Fortune MD , Sparkes D , Curran MD , Zhang H , Jalal H . 2021. Specificity and positive predictive value of SARS-CoV-2 nucleic acid amplification testing in a low-prevalence setting. Clin Microbiol Infect 27:469. doi:10.1016/j.cmi.2020.10.003 PMC7554481 33068757

[14] Pollock NR , Lee F , Ginocchio CC , Yao JD , Humphries RM . 2021. Considerations for assessment and deployment of rapid antigen tests for diagnosis of coronavirus disease 2019. Open Forum Infect Dis 8:ofab110. doi:10.1093/ofid/ofab110 34258309 PMC7989197

[15] U.S. Food and Drug . 2023 SARS-CoV-2 viral mutations: impact on COVID-19 tests | FDA. Available from: https://www.fda.gov/medical-devices/coronavirus-covid-19-and-medical-devices/sars-cov-2-viral-mutations-impact-covid-19-tests

[16] Guglielmi G . 2020. Fast coronavirus tests: what they can and can’t do. Nature 585:496–498. doi:10.1038/d41586-020-02661-2 32939084

[17] Adamson B , Sikka R , Wyllie AL , Premsrirut P . 2022. Discordant SARS-CoV-2 PCR and rapid antigen test results when infectious: a december 2021 occupational case series. Infect Dis. doi:10.1101/2022.01.04.22268770

[18] Dinnes J , Deeks JJ , Berhane S , Taylor M , Adriano A , Davenport C , Dittrich S , Emperador D , Takwoingi Y , Cunningham J , Beese S , Domen J , Dretzke J , Ferrante di Ruffano L , Harris IM , Price MJ , Taylor-Phillips S , Hooft L , Leeflang MM , McInnes MD , Spijker R , Van den Bruel A , Cochrane COVID-19 Diagnostic Test Accuracy Group . 2021. Rapid, point-of-care antigen and molecular-based tests for diagnosis of SARS-CoV-2 infection. Cochrane Database Syst Rev 3:CD013705. doi:10.1002/14651858.CD013705.pub2 32845525 PMC8078202

[19] Sakthivel D , Delgado-Diaz D , McArthur L , Hopper W , Richards JS , Narh CA . 2021. Point-of-care diagnostic tools for surveillance of SARS-CoV-2 infections. Front Public Health 9:766871. doi:10.3389/fpubh.2021.766871 34900912 PMC8655681

[20] US Health and Human Services, Administration for Strategic Preparedness and Response . 2022 COVID-19 test to treat. Available from: https://aspr.hhs.gov/TestToTreat/Pages/default.aspx#:~:text=The%20Biden%2DHarris%20Administration%20launched,lifesaving%20treatment%20for%20COVID%2D19

[21] Cue health to launch direct-to-consumer virtual health platform featuring its COVID-19 self-test trusted by google, mayo clinic, the NBA, and MLB. 2021. Available from: https://www.prnewswire.com/news-releases/cue-health-to-launch-direct-to-consumer-virtual-health-platform-featuring-its-covid-19-self-test-trusted-by-google-mayo-clinic-the-nba-and-mlb-301419256.html

